# RADAR – Radiomics on aSDH: predicting outcome with surface area

**DOI:** 10.1007/s00701-024-06408-0

**Published:** 2025-01-20

**Authors:** Antonia Richter, Johannes Wach, Alim Basaran, Johannes Kasper, Florian Wilhelmy, Tim Wende, Felix Arlt, Ági Güresir, Erdem Güresir, Martin Vychopen

**Affiliations:** Department of Neurosurgery, University Hospital Leipzig, 04103 Leipzig, Germany

**Keywords:** Radiomics, Acute subdural hematoma, Surface area, Outcome

## Abstract

**Background:**

Acute subdural hematoma is a critical condition, leading to significant morbidity and mortality. Despite advancements in surgical techniques, a portion of patients only show limited clinical improvement post-evacuation. Surgical intervention decisions are critically important, as they can either improve or worsen a patient’s condition. Radiomics offers significant potential by extracting complex patterns from digital medical images and transforming them into high-dimensional data that reflect the underlying pathophysiology. By integrating Radiomics with individual patient characteristics, we can develop decision support models. This study aims to analyze radiomic parameters of aSDH to determine whether they support the decision to proceed with urgent surgery or opt for a conservative approach. We hypothesized that surface area could be a significant predictor of neurological outcome such as maintaining independent mobility (mRS ≥ 3) and survival rates.

**Methods:**

This retrospective study involved radiomic analysis according to neurological outcome and survival. Radiomic parameters were measured using 3D Slicer software. Statistical analyses explored correlations, employing AUC-analysis and Kaplan-Meier survival.

**Results:**

Our findings revealed significant correlations between hematoma and surface area with poorer neurological prognosis. Further subgroup analysis showed surface area as a significant predictor for poorer outcomes in patients undergoing craniotomy (*p* = 0.006 in univariant- and *p* = 0.020 in multivariant analysis). In the total cohort, among conservatively managed and craniotomy subgroups, survival analysis highlighted an advantageous survival for patients exhibiting smaller surface areas (< 339.50 cm^2^).

**Conclusions:**

Especially in craniotomy patients, surface area emerged as a possible predictor for neurological outcome and survival.

## Introduction

### Background

Acute subdural hematoma (aSDH) is a common neurosurgical condition concerning patients with traumatic brain injury frequently accompanied by considerable morbidity and mortality [7, 32, 35, 42, 57]. Therefore, the relevance of aSDH should not be underestimated in clinical practice. To attain favorable outcome, emergent surgical evacuation of hematoma through craniectomy or craniotomy according to presented criteria in Traumatic Brain Injury guideline is often performed in patients with aSDH [8, 55]. Nevertheless, the rate of complications can reach up to 10.0–40.4% [25]. Even after the effective evacuation of aSDH, around one-third of patients exhibit no clinical improvement [59].

Experimental studies focused on traumatic brain injury (TBI) models have shown cortical damage following blood effusion in animals, supporting the hypothesis that hematoma volume is not the sole determinant of parenchymal damage [5]. In this context, parameters like surface area or Feret diameter may offer a more accurate assessment and better account for potential cortical damage.

### Clinical relevance

Acute subdural hematoma represents an acute intracranial lesion which could lead to a rapid elevation of intracranial pressure. Due to the presence of aSDH, many patients experience a compromised health status. The consideration of surgical treatment may either exacerbate or potentially benefit their condition. Hence, it would be advantageous to have a parameter for guiding the decision-making process, delineating which patients are most suitable for conservative management and which necessitate surgical intervention.

### Radiomics

Radiomics is a new field in medicine that is gaining increasing importance in both clinical and experimental settings. It seeks to derive quantitative and ideally reproducible data from diagnostic images. This encompasses intricate patterns that may be challenging for the human eye to identify [41]. It is possible to extract numerous quantitative features from computed tomography (CT), magnetic resonance tomography (MRT), or positron emission tomography (PET) images. Radiomics involves converting digital medical images into high-dimensional data because biomedical images are believed to encapsule information reflective of their underlying pathophysiology. These relations can be unveiled through quantitative image analysis. Radiomics aims to develop decision support tools by integrating radiomic data with other available patient characteristics, thereby enhancing the effectiveness of decision support models [17].

### Study goals

To our knowledge Radiomics quantitative analysis of aSDH has not been performed yet. Our objective was to assess whether radiomic parameters can anticipate the neurological outcome. The aim is to identify a patient cohort potentially profiting from conservative treatment or surgical intervention. We conducted this study to identify potential radiomic risk factors for aSDH that are correlated with the occurrence of poor neurological outcome.

## Methods

### Research cohort and data collection

In this retrospective study we searched our institutional database and analyzed a cohort of 178 patients with aSDH between January 2015 and December 2022. Acute subdural hematoma was radiologically characterized by the presence of a hyperdense subdural collection. In this study, we included patients who initially presented with an acute subdural hematoma who were older than 18 years. We also considered patients whose acute subdural hematoma subsequently progressed to a chronic condition, as represented by the subgroup that underwent burr-hole trephination. However, patients admitted to our clinic with primarily chronic subdural hematoma were excluded, as their pathophysiology and pathogenesis differ significantly from those of acute cases. Patients with severe penetrating brain injuries and high-speed impact polytraumas were excluded as well [39, 40]. The approval for this study was granted by the Clinical Ethics Committee of the University of Leipzig (362/23-ek).

We investigated the following parameters: patient characteristics (age, sex etc.), date of admission, timing of surgery, APACHE-Score after surgery, cardiac comorbidities, admission status according to Glasgow Coma Scale (GCS) and pupillomotor skills, anticoagulation status, method of surgical treatment (conservative vs. craniotomy vs. craniectomy vs. burr-hole trephination), preoperative coagulation and inflammatory status, the use of substitution therapy during surgery and revision surgery rate.

For radiological measurements the initial CT scan upon admission was utilized. Neurological outcomes were assessed based on modified Rankin Scale (mRS) when discharged, as well as during the three-months, six-months and twelve-months follow-up assessments. The follow-up mRS-Scores were determined through evaluations conducted during follow-up visits to our hospital. The mRS-Score at discharge was again stratified into good and poor outcome. In the case of acute subdural hematoma, we are frequently confronted with critically elevated intracranial pressure often leading to confined neurological status, therefore we decided to dichotomize the mRS-Score in 0–3 (good outcome) and 4–6 (poor outcome) as other randomized controlled trials addressing similar high intracranial pressures performed the same dichotomization [24, 52].

## Image analysis

Admission CT images were obtained at our institution. We also acquired the initial CT scans from referring hospitals. All scans were performed within 24 h after head trauma. We measured hematoma associated radiomic parameters with open-source software 3D Slicer (version 5.2.2).

**Fig. 1:**
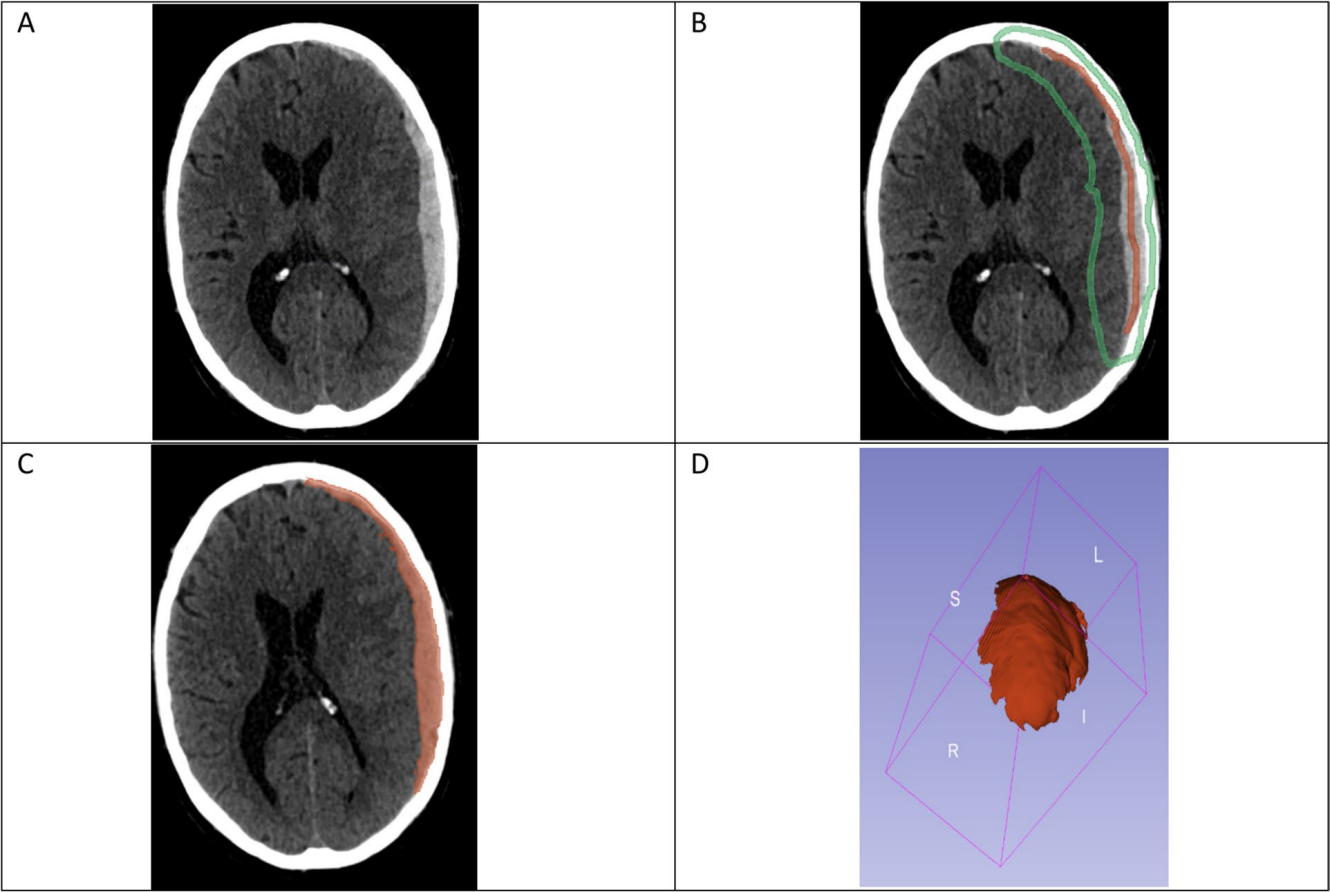
Segmentation in 3D slicer. **A** Unedited CT-scan. **B** Marking acute subdural hematoma in red and surrounding areas in green in multiple image planes and all three axes. **C** Semiautomated two-dimensional reconstruction of hematoma. **D** Rotatable three-dimensional reconstruction of hematoma that is used for calculating wanted parameters

After looking through admission CT scans, we exported them as DICOM data and imported them into 3D Slicer. First, we segmented the hematoma in segment editor. For this purpose, we used option ´growing seeds`, marking sections of aSDH as hematoma and the surrounding tissue (like brain or skull) as background to differentiate them. After delineating the subdural hematoma in 3D-Slicer, the software performed a semi-automated segmentation. Following this, the investigator (AR) manually refined the segmented areas, which were subsequently verified by two independent supervisors (MV, EG) to minimize any potential cognitive bias. This approach ensured that the process involved both semi-automated and manual segmentation, allowing us to achieve the highest possible precision in line with practical guides that address neurosurgical segmentation [27]. After manual refinement, 3D Slicer performed a three-dimensional reconstruction of the hematoma that can be seen in ´3D window`. Detailed information can be found in Fig. 1.

For calculations we used Labelmap Statistics that are calculated using the binary labelmap representation of the segment. 3D Slicer will quantify parameters only using the already segmented part of the CT scan. In our study Feret diameter, volume, and surface area were measured. Surface area represents the cumulated surface of the aSDH and not solely the part in contact with brain parenchyma but also with the skull.

### Statistical analysis

Data analysis was conducted utilizing IBM SPSS Statistics (version 29, IBM Corp.). Patients were divided into two groups depending on whether they underwent surgery or were treated conservatively. Three surgical categories were differentiated: craniotomy, craniectomy and burr-hole trephination. Data will be presented by using mean ± standard deviations for continuous variables such as age, GCS, and modified Rankin Scale and as percentages for discrete variables such as sex and mortality. For categorial parameters we examined the variables in a contingency table using either Fisher´s exact test or the chi-squared test, as deemed suitable. Statistical significance was defined as a p-value ≤ 0.05 and all tests were two-sided.

A Receiver operating characteristic (ROC) analysis was performed since it serves as a valuable method of model predictions. ROC curves are employed to assess the discriminative capability of several statistical approaches that integrate different test results and clues for predictive goals (e.g. whether a patient will profit from a certain treatment) [21]. The total area under a specific ROC curve (known as AUC) constitutes a crucial statistic, indicating the probability of correct prediction when observing a test variable. The AUC calculation was utilized to assess radiomic parameters (volume, surface area and Feret diameter) as predictors for poor outcome in patients with aSDH. Using ROC analysis, cut-off values were determined for all three parameters, followed by dichotomization into good and poor outcome [47]. Univariant analysis was performed based on the dichotomized values of our radiomic parameters in relation to the mRS-Score at the time of discharge. The same mRS-Scores were used to perform multivariant regression analysis considering our radiomic parameters and further outcome predictors described in other studies like age, pupillary abnormities and GCS [2, 23, 45, 50]. We used the calculated cut-off values from the initial ROC analysis to dichotomize Feret diameter, surface area, and volume. Age was categorized into groups above and below equal 70, as outlined in similar studies [50, 51]. The GCS-Score was divided into outcomes greater and smaller equal eight according to Younsi et al. [62]. Pupillomotor skills were classified into isocor and anisocor.

Additionally, we used the mRS-Scores of the follow up assessments to perform a Kaplan-Meier Analysis to examine survival based on the identified cut-off values of the radiomic parameters. To compare the groups, we conducted a Log Rank (Mantel-Cox) Analysis.

## Results

### Patient characteristics

In total, 178 patients were qualified for this study. We saw a male dominant cohort (102:76) with a median age at diagnosis of 70.41 ± 15.908 (range 20–93). In our cohort, 131 patients were operated, where 47 patients obtained conservative treatment. Twelve patients underwent burr-hole trephination, 64 craniotomy and 55 craniectomy where the mean surgery time in all three procedures was 87.34 min (± 35.858). 22 patients had to get revision surgery. Further parameters including GCS and pupillomotor skills on admission, history of anticoagulation, mortality, use of substitution therapy during surgery, postoperative APACHE-Score, cardiac comorbidities, and mRS-Scores are described in Table 1. Based on Glasgow Coma Scale the brain injury was distinguished in mild (GCS 15 − 13), moderate (12 − 9) and severe (8 − 3) according to Brain Injury Association of America [18]. The mRS-Scores were assessed on discharge day, three months, six months, and twelve months after discharge. They were then dichotomized in good (mRS 0–3) and poor (mRS 4–6) neurological outcome.

**Table 1:** Patient characteristics

patient characteristics	all patients	surgical patients	conservative patients	*p*-value
number of patients	178	131 (26.4%)	47 (73.6%)	−
age mean	70 (± 15.908)	68.53 (± 15.220)	75.64 (± 16.767)	n.s.
sex (male : female)	102 : 76 (57.3% : 42.7%)	81 : 50 (61.8% : 38.2%)	21 : 26 (44.7% : 55.3%)	n.s.
GCS (valid *n* = 120) -> 15−13 -> 12−9 -> 8 − 3	-> 37 (30.8%) -> 23 (19.3%) -> 60 (50.0%)	-> 22 (23.1%) -> 20 (21.2%) -> 53 (55.9%)	-> 15 (60.0%) -> 3 (12.0%) -> 7 (28.0%)	n.s.
intact pupillomotor skills (valid *n* = 145)	89 (61.4%)	60 (54.5%)	29 (82.9%)	0.003
history of anticoagulant/antiplatelet medication (valid *n* = 173)	87 (50.3%)	61 (48.4%)	26 (55.3%)	n.s.
pre-existing cardiac illnesses (valid *n* = 173)	121 (69.9%)	84 (66.1%)	37 (80.4%)	n.s.
substitution of coagulation factors (valid *n* = 178)	58 (32.6%)	44 (33.6%)	14 (29.8%)	n.s.
epileptic seizures (valid *n* = 178)	50 (28.1%)	42 (32.1%)	8 (17.0%)	n.s.
revision surgery rate (valid *n* = 136)	−	22 (16.8%)	−	−
mortality (valid *n* = 187)	41 (23.0%)	35 (26.7%)	6 (12.8%)	n.s.
APACHE-Score median (valid *n* = 165)	14	15	12	−
mRS at discharge (valid *n* = 177) -> 0–3 -> 4–6	-> 67 (37.8%) -> 110 (62.1%)	-> 34 (26.1%) -> 96 (73.8%)	-> 33 (70.2%) -> 14 (29.7%)	−
mRS after 3 months (valid *n* = 128) -> 0–3 -> 4–6	-> 63 (49.2%) -> 65 (50.8%)	-> 40 (40.4%) -> 59 (59.6%)	-> 23 (79.3%) -> 6 (20.6%)	−
mRS after 6 months (valid *n* = 93) -> 0–3 -> 4–6	-> 32 (34.4%) -> 61 (65.6%)	-> 24 (30.3) -> 55 (69.6)	-> 8 (57.1%) -> 6 (42.9%)	−
mRS after 12 months (valid *n* = 70) -> 0–3 -> 4–6	-> 21 (30.1%) -> 49 (70.0%)	-> 20 (32.7%) -> 41 (67.3%)	-> 1 (11.1%) -> 8 (88.9%)	−
surface area in cm^2^ mean (valid *n* = 178)	407.84 (± 375.33)	447.93 (± 403.40)	296.08 (± 254.58)	< 0.001
volume in cm^3^ mean (valid *n* = 177)	89.59 (± 63.19)	102.36 (± 59.92)	54.27 (± 58.91)	< 0.001
Feret diameter in cm mean (valid *n* = 178)	14.16 (± 2.9)	14.61 (± 2.73)	12.9 (± 2.92)	0.002

*APACHE-Score* (Acute Physiology And Chronic Health Evaluation-Score), *GCS* (Glasgow Coma Scale), *mRS* (modified Rankin Scale), *n.s.* (not significant)

### Radiomics

**Fig. 2:**
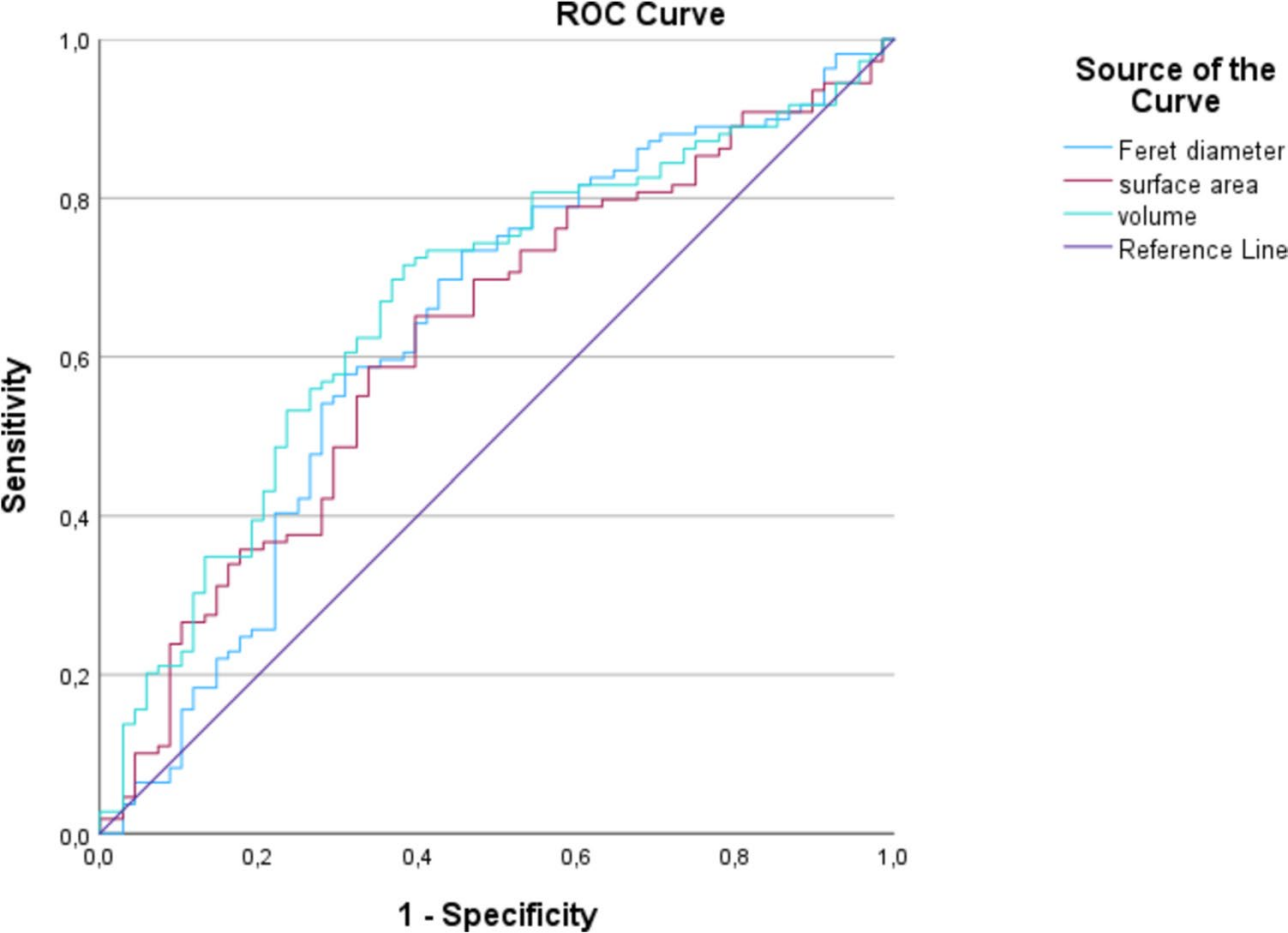
Receiver operating curve representing sensitivity against 1-specificity. Feret diameter is displayed by the blue, volume by the turquoise, and surface area by the lilac curve. All parameters show an AUC above 0.6

Our results show that volume, surface area and Feret diameter demonstrate a correlation with poorer neurological prognosis. Volume displays an AUC of 0.668 with a cut-off value of 73.58 cm^3^ meaning that aSDH with larger volumes are more likely to be associated with poor neurological outcome (mRS 4–6). Surface area and Feret diameter show the AUC of 0.623 and 0.632 with cut-off values of 339.50 cm^2^ and 14.59 cm. For detailed information see Table 2; Fig. 2.

**Table 2:** Receiver operating curve analysis

ROC analysis	AUC	CI 95%	cut-off
surface area	0.623	0.539–0.708	339.50 cm^2^
volume	0.668	0.587–0.750	73.58 cm^3^
Feret diameter	0.632	0.546–0.719	14.59 cm

Presented is the AUC (area under the curve), the CI 95% (95% Confidence Interval) and the cut-off value for surface area, volume and Feret diameter

### Total cohort

We then performed an univariant analysis, dichotomizing the radiomic parameters based on their cut-off values. Feret diameter, volume and surface area exhibited statistical significance. Patients with a surface area exceeding 339.50 cm^2^ faced a 2.459-fold increased risk of experiencing an unfavorable outcome. Similarly, individuals with a volume surpassing 73.58 cm^3^ had a 3.568-fold elevated risk. Analogously, Feret diameters exceeding 14.58 cm were associated with a 2.556-fold increased risk of poor neurological outcome. See Table 3.

**Table 3:** Univariant analysis for total cohort

univariant analysis	*p*-value	CI 95%	risk
surface area	0.005	1.323–4.569	2.459
volume	< 0.001	1.889–6.739	3.568
Feret diameter	0.003	1.374–4.754	2.556

Presenting *p*-values of the radionomic parameters dichotomized by cut off values in relation to mRS-Score at discharge, the consequently elevated risk for poorer neurological outcome and their CI 95% (95% confidence interval)

### Subgroups according to therapy of choice

Subsequently we divided our cohort into four groups according to performed treatment: conservative (*n* = 47), burr-hole trephination (*n* = 12), craniotomy (*n* = 64) and craniectomy (*n* = 55). We conducted univariant analysis considering the dichotomized mRS-Score at discharge in relation to the radiomic parameters divided according to our calculated cut-off values in the ROC analysis.

In the category of conservatively treated patients, a correlation between volume and unfavorable outcome was observed with a p-value of 0.025. This indicates that individuals in this subgroup exhibiting aSDH with volumes exceeding 73.58 cm^3^ faced a 5.6-fold increased risk of poorer prognosis. Neither surface area nor Feret diameter could be determined as predictive outcome parameters. Looking at patients treated with burr-hole trephination there was no significant predictor of neurological outcome.

When considering individuals that underwent craniotomy, surface area could be identified as a significant predictor for unfavorable neurological outcome presenting a p-value of 0.006. This implies that patients in this subgroup exhibiting aSDH with a larger surface area than 339.50 cm^2^ are exposed to a 4.952-fold higher risk to attain unfavorable neurological outcome. Also, volume and Feret diameter show statistical tendency with p-value of 0.057 and 0.051.

 In patients undergoing craniectomy there was no significant predictor for neurological outcome. For further information refer to Table 4.

**Table 4:** Univariant analysis for each subgroup

		*p*-value	95% CI	risk
no intervention				
	surface area	0.324	0.541–7.388	2.000
	volume	0.025	1.360–23.059	5.600
	Feret diameter	0.182	0.728–9.764	2.667
burr-hole trephination				
	surface area	0.318	0.004–3.525	0.111
	volume	0.455	0.010–5.985	0.250
	Feret diameter	0.455	0.010–5.985	0.250
craniotomy				
	surface area	0.006	1.594–15.382	4.952
	volume	0.051	1.046–9.529	3.157
	Feret diameter	0.057	1.076–9.815	3.250
craniectomy				
	surface area	0.643	0.318–13.511	2.071
	volume	0.342	0.443–19.129	2.912
	Feret diameter	1.000	0.197–8.502	1.294

Presenting *p*-values of the radionomic parameters dichotomized by cut off values in relation to mRS-Score at discharge, the consequently elevated risk for poorer neurological outcome and their CI 95% (95% confidence interval)

### Multivariant regression analysis

After conducting an univariant analysis, testing of multivariant prediction models was performed. The results align with the results from univariant analysis. In the conservatively treated subgroup, the multivariate analysis identified volume as a significant outcome predictor with a p-value of 0.044, which reinforces our previous findings. Additionally, GCS emerged as a significant factor with a p-value of 0.032.

Considering the limited number of patients who underwent burr-hole trephination (*n* = 12), performing a multivariate analysis could introduce substantial bias into the findings. Consequently, we omitted this calculation.

When looking at patients who underwent craniectomy, surface area stands out as significant with a *p*-value of 0.02. Finally, for patients with decompressive craniectomy no parameter emerged as a significant outcome predictor confirming our results from the univariant analysis. Table 5 provides further information.

**Table 5:** Multivariant analysis for conservative and craniotomy patients

		*p*-value	95% CI
conservative subgroup			
	volume	0.044	1.076–215.372
	Feret diameter	0.911	0.007–80.142
	surface area	0.999	–
	age	0.528	0.118–69.984
	GCS	0.032	1.326–524.867
	pupillomotor skills	0.542	0.072–150.164
craniotomy subgroup			
	volume	0.461	0.021–5.754
	Feret diameter	0.931	0.015–98.488
	surface area	0.020	1.535–146.545
	age	0.394	0.329–16.811
	GCS	0.986	0.063–15.213
	pupillomotor skills	0.999	–

Presenting *p*-values of radiomic parameters and possible outcome predictors in relation to dichotomized modified Rankin Scale at discharge and their 95% CI (95% confidence interval)

### Survival

As mentioned above, the univariant analysis refers to the day of discharge and does not disclosure long-term outcome. Hence, we decided to perform a Kaplan-Meier Analysis to display survival. Since we could identify volume and surface area as significant outcome predictor in some of the subgroups, we decided to focus on surface area because volume is already described as a significant outcome predictor in other sources [6, 15, 53, 58]. We were presented with significant results for the total cohort, the conservative and craniectomy subgroup, but not for the burr-hole trephination and craniectomy subgroup.

A) Total cohort

**Fig. 3:**
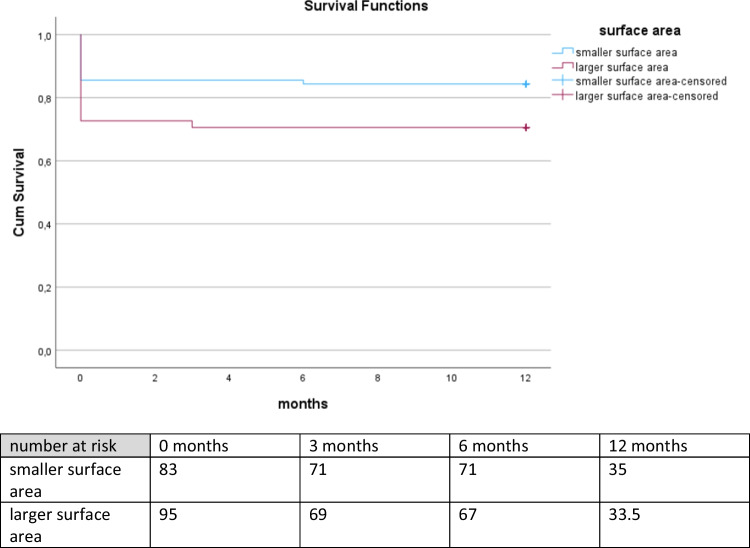
Kaplan-Meier survival for total cohort according to surface area. The Kaplan-Meier curve depicts the survival for twelve months of all patients with surface area above (lilac curve) versus below (turquoise curve) the cut-off value for surface area. Also shown is the number of patients at risk on the day of discharge, three months, six months and twelve months after discharge representing the patients that are still alive

The Kaplan-Meier Analysis showed a significant advantage in survival for patients displaying a surface area lower than 339.50 cm^2^ with a Log rank (Mantel Cox) = 0.029. Additionally, the number of patients at risk at discharge and during the follow-up examinations is presented. The numbers at three months, six months and twelve months after discharge are higher in the group with smaller surface area representing those patients that survived. Refer to Fig. 3.

B) No intervention

**Fig. 4:**
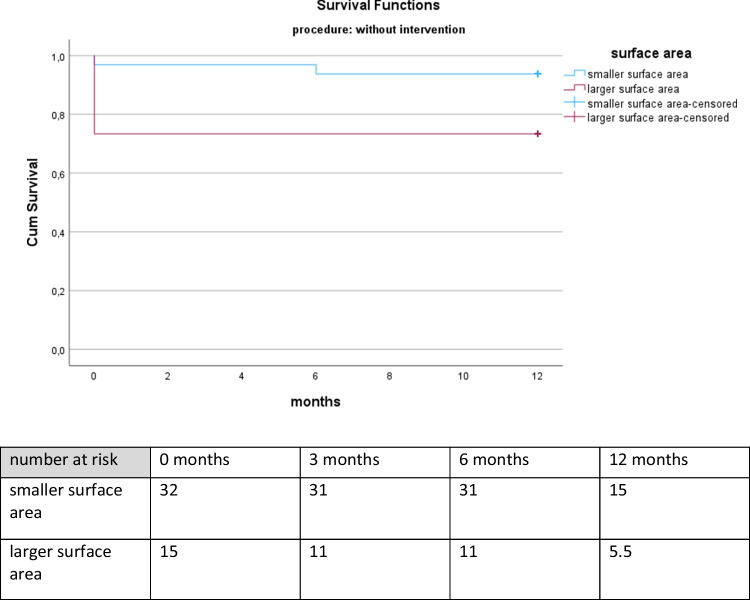
Kaplan-Meier survival for patients treated conservatively according to surface area. The Kaplan-Meier curve depicts the survival for twelve months of patients treated conservatively with surface area above (lilac curve) versus below (turquoise curve) the cut-off value for surface area. Also shown is the number of patients at risk on the day of discharge, three months, six months and twelve months after discharge representing the patients that are still alive

The Kaplan Meier Analysis showed a difference in survival for the two surface area groups. Patients presenting a surface area lower than 339.50 cm^2^ were more likely to survive with a Log rank (Mantel Cox) = 0.049. For further information see Fig. 4.

C) Craniotomy

**Fig. 5:**
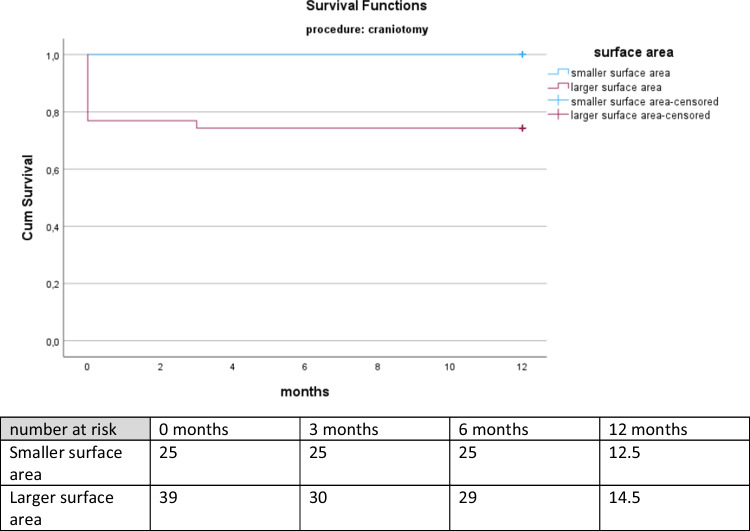
Kaplan-Meier survival for patients undergoing craniotomy according to surface area. The Kaplan Meier curve displays survival for twelve months of patients undergoing craniotomy with surface area above (lilac curve) versus below (turquoise curve) the cut-off value for surface area. Also shown is the number of patients at risk on the day of discharge, three months, six months and twelve months after discharge representing the patients that are still alive

The Kaplan-Meier Analysis demonstrated a notable survival advantage in the patient group with a surface area lower than 339.50 cm^2^, as evidenced by a Log rank (Mantel Cox) = 0.006, indicating a statistically significant difference. No deaths were observed in this subgroup during the study period. See Fig. 5.

## Discussion

Our cohort was male dominant with an average age of 70.41 (± 15.908). 69.9% suffered from a cardiac illness and 50.3% were anticoagulated. So, we faced a typical cohort for aSDH [38, 43]. It was divided into four groups based on therapy approach. 55 patients underwent craniectomy, 64 patients craniotomy, 12 patients burr hole trephination and 47 patients were treated conservatively. The optimal clinical outcome is believed to occur when aSDH is allowed to undergo chronification because cSDH evacuation is associated with better outcomes [1, 11, 20, 30, 34, 54]. However, given that numerous patients require immediate surgery and cannot afford to wait for chronification, having a prognostic parameter for distinguishing between good and poor outcomes in cases of emergent evacuation could sharpen the indication criteria. Radiomics could be an instrument to achieve this objective. Looking at the generation of surface maps directly from biomedical imaging data, 3D Slicer exhibits advantages over other open-source programs. It displays the ability to load image series containing a greater number of images and generate more precise surface maps [10, 37, 46, 49].

### Neurological outcome

The results of univariate and multivariate analyses emerged consistent with each other. When treated conservatively we could identify volume as an outcome predictor in both analyze approaches, confirmed through the results of previous published studies [6, 15, 53, 58]. Additionally, in multivariant analysis GCS score proved to be significant what is also described in other studies [50, 51]. In the subgroup undergoing burr-hole trephination, for univariant analysis no significant outcome predictor was identified. Multivariant analysis was not performed due to deficient number of patients. We also could not find an outcome predictor in the craniectomy subgroup which was shown in uni- and multivariant analysis. This result may be attributed to the fact that craniectomy is typically undertaken as an urgent, life-saving procedure, introducing a myriad of additional factors that contribute to the complexity of prognosis assessment [9, 28, 48]. Hence, in our limited cohort radiomics analysis might not be sufficient for outcome determination. When looking at patients who underwent craniotomy, we could identify surface area as a significant predictor for poor neurological outcome in both analysis approaches.

**Fig. 6:**
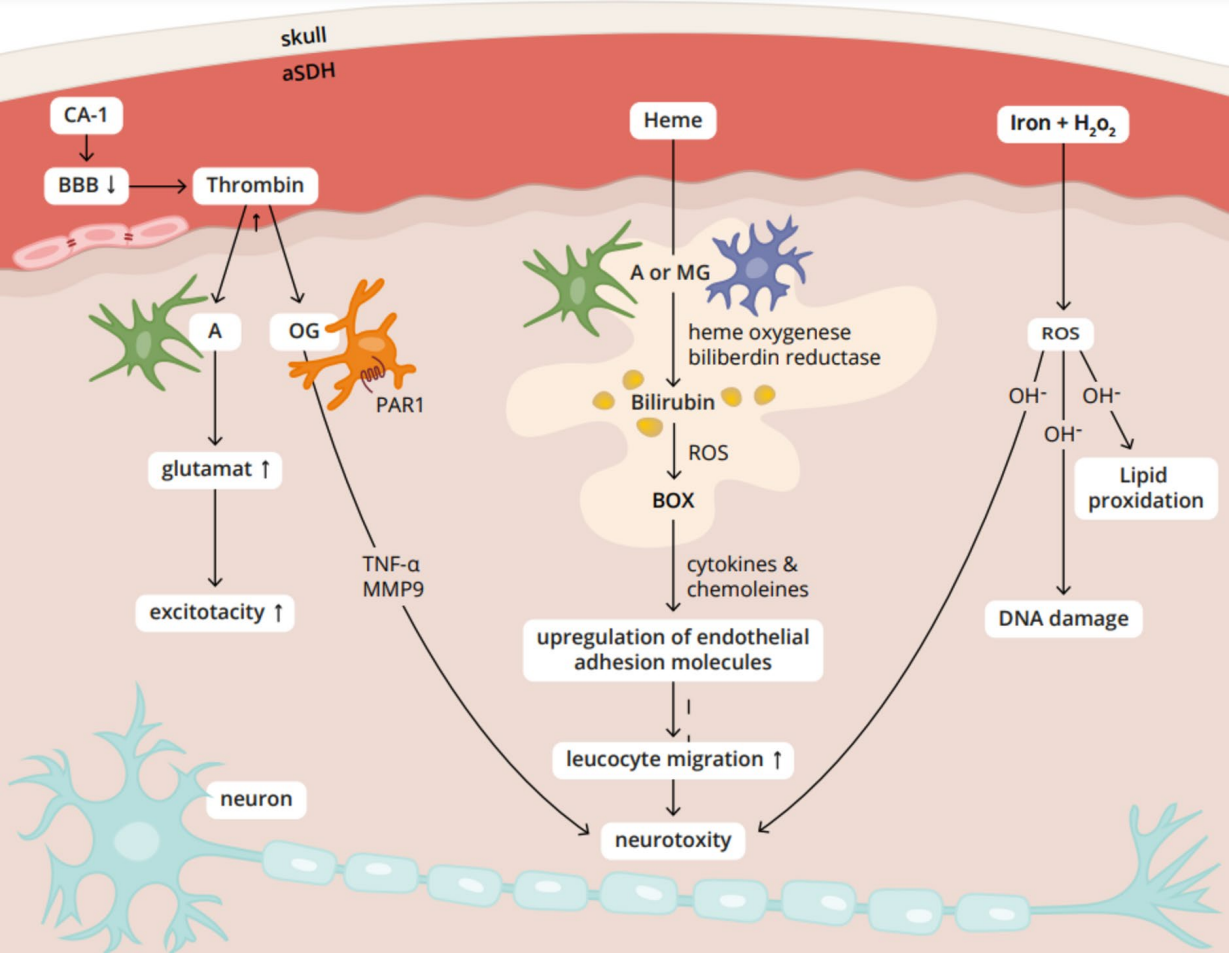
Interaction between acute subdural hematoma and brain surface. A: astrocyte. aSDH: acute subdural hematoma. BBB: blood-brain barrier. BOX: bilirubin oxidation products. CA-1: carbonic anhydrase I. MG: microglia. MMP9: matrix metalloproteinase-9. OG: oligodendrocytes. PAR1: protease activated receptor 1. ROS: reactive oxygen species. TNF-α: tumor necrosis factor alpha. CA-1 from lysed erythrocytes lowers the blood-brain barrier, making it easier for thrombin to cross. This can subsequently increase glutamate efflux in astrocytes, leading to a lower epileptic threshold. Additionally, thrombin can bind to the protease activated receptor 1 (PAR 1) in oligodendrocytes, stimulating the release of MMP9 and TNF-α, which can have neuro-damaging effects. Heme from lysed erythrocytes is converted into bilirubin by heme oxygenase-1 and biliverdin reductase in oligodendrocytes and microglia, which is later transformed into bilirubin oxidation products (BOX) by reactive oxygen species leading to a release of cytokines and chemokines. This enables the upregulation of endothelial adhesion molecules resulting in an increased leucocyte migration. Iron from the acute subdural hematoma can react with H_2_O_2_ and generate reactive oxygen species. These can cause harmful effects such as DNA damage and lipid peroxidation in neurons

To spare patients from avoidable surgeries and their potential consequences, there should be a predictor to estimate neurological prognosis since acute subdural hematoma is not solely characterized by a compression effect. There is also direct contact between blood components and the cerebral cortex, leading to further complex consequences [60]. For instance, the presence of carbonic anhydrase I (CA-1) released from lysed erythrocytes has the potential to elevate the blood-brain barrier permeability, resulting in vasogenic edema, microglial activation, and neuronal apoptosis [14, 19, 29]. Furthermore, heme and its byproducts activate microglia and astrocytes, leading to the infiltration of leukocytes and production of cytokines, thereby intensifying injury [36]. Iron may initiate oxidative damage to the brain and the direct interaction of thrombin with brain cells can instigate cell death [13, 33, 61]. Also, Thrombin increases glutamate efflux [26, 44] which could lower epileptic threshold [12] representing an additional risk factor for poorer neurological outcome. Further information can be found in Fig. 6. In addition, we could investigate further cell death mechanisms due to subdural hemorrhage irritating the brain surface like caspase activation. Acute subdural hematoma (aSDH) leads to a rapid increase in intracranial pressure (ICP) and a decrease in tissue oxygen concentration, triggering ischemic events that result in apoptotic caspase dependent cell death [3].

Hence, it may not be the size of the hematoma that is crucial but also its contact surface with the cortex. Since volume was not significant in the conservative subgroup, we assume that especially narrow and wide hematomas could pose a risk for poor neurological outcome. This risk could be assessed using the radiomic parameter surface area for patients undergoing craniotomy. This implies that hematomas exhibiting a surface area less than 339.50 cm^2^ may warrant conservative management, while those surpassing this threshold should undergo craniotomy.

### Survival

Our analysis revealed that surface area could serve as a survival predictor for aSDH. We observed a significant difference in survival between patients with a surface area above and below the determined cut-off value. This significance was evident not only in the total cohort but also in the no intervention and craniotomy subgroups. However, there was no significant difference in the burr-hole trephination and craniectomy subgroups, which confirms our previous results. The most pronounced difference was observed in the time until hospital discharge, indicating that this period is particularly crucial for survival in patients with aSDH.

### Limitations

Limitations are associated with this study, including the immanent constraints of a small cohort size due to the uncommonness of this disease which limits our statistical power. Given the variance in severity of aSDH, our cohort required division into four subgroups to depict the different therapeutic regimens. Because of this, we were not able to further split our cohort in a development and validation cohort for more predictive power as other studies were capable of doing so [22].

Because this study was conducted in a retrospective design, initial therapy decision was not based on radiomic parameters. This could lead to selection bias since the treatment structure in Germany relies heavily on outline clinics where patients with less severe clinical symptoms are presented. Therefore, numerous patients who would typically fall under the conservative subgroup were not treated at our hospital. Consequently, we must assume that the percentage of patients in the conservative subgroup would likely be higher in studies conducted prospectively. Additionally, no randomization was carried out and the preoperative neurological baseline function prior to trauma was often not documented.

Furthermore, the outcome is not only influenced by the hematoma itself but also by primary brain injuries such as contusions, lacerations, and diffuse axonal injury, leading to a secondary damage like high intracranial pressure, reduced blood flow and hypoxia [4, 31, 56, 60]. The pathomechanism illustrated in Fig. 6 represents an attempt to elucidate the interaction between acute subdural hematoma and the brain. However, as it is not standard practice to examine cerebrospinal fluid in patients with aSDH, we were unable to perform a statistical analysis of the pathophysiological mechanism proposed by us. These factors were especially a main limitation in analysis of craniectomy patients. This might be a crucial part for future prospective studies covering this topic.

A considerable number of patients were lost at the follow up assessments (27.68% for the three-months, 47.46% for the six-months and 60.45% for the twelve-months follow-up) which might be due to treatment structure in Germany. Many follow-up assessments are carried out at outpatient clinics and through teleradiology, which means that patients may not return to our hospital for further visits. This is especially valid for patients who experience favorable neurological outcomes, as they are less likely to come back for follow-up appointments. This challenges the ability to provide significant insights into long-term outcomes. For exact evaluation of our results, multicentric or even prospective setting might be necessary.

## Conclusion

In summary our study faced a typical cohort for aSDH. Neurological outcomes were assessed using modified Rankin Scale, with larger hematoma volumes and surface areas, and Feret diameters linked to poorer outcomes. Volume > 73.56 cm^3^, surface area > 339.50 cm^2^ and Feret diameter > 14.59 cm increased the risk of poor outcomes. Relevant findings include the identification of volume as an outcome predictor in conservatively treated patients. For patients undergoing craniotomy, surface area emerged as a significant predictor for poor neurological prognosis. No significant predictors were found for the burr-hole trephination or craniectomy subgroups.

When focusing on survival, the Kaplan-Meier analysis showed better survival for patients with surface areas < 339.50 cm^2^ in the total cohort, as well as in the conservative and craniotomy subgroups. Overall, hematoma size is crucial in predicting neurological outcomes and survival in acute subdural hematoma patients. Nevertheless, further research, e.g. in a prospective study with a larger cohort is essential to thoroughly investigate this topic. Non-invasive methods such as hyperspectral imaging (HSI) could be a potential approach to explore this further and provide more insights into the human brain since HSI can be utilized as a non-invasive technique to visualize metabolic processes in the brain [16].

## Data Availability

Data is available on request by corresponding author. Supported by the Open Access Publishing Fund of Leipzig University.

## Abbreviations

APACHE -Score: Acute Physiology And Chronic Health Evaluation-Score
aSDH: acute subdural hematoma
AUC: area under the curve
cSDH: chronic subdural hematoma
CI: confidence interval
CT: computed tomography
GCS: Glasgow Coma Scale
HIS: hyperspectral imaging
mRS: modified Rankin Scale
MRT: magnetic resonance tomography
n.s.: not significant
PET: positron emission tomography
ROC: receiver operating curve
TBI: traumatic brain injury
